# Headstrong intervention for pediatric migraine headache: a randomized clinical trial

**DOI:** 10.1186/1129-2377-15-12

**Published:** 2014-02-28

**Authors:** Michael A Rapoff, Mark Connelly, Jennifer L Bickel, Scott W Powers, Andrew D Hershey, Janelle R Allen, Cynthia W Karlson, Catrina C Litzenburg, John M Belmont

**Affiliations:** 1 University of Kansas Medical Center, Department of Pediatrics, 3901 Rainbow Boulevard, Kansas City, KS 66160-7330, USA; 2 Children’s Mercy Hospital, 2401 Gillham Road, Kansas City, MO 64108, USA; 3 Cincinnati Children’s Hospital Medical Center, 3333 Burnet Avenue, Cincinnati, Ohio 45229, USA; 4 University of Mississippi Medical Center, 2500 North State Street, Jackson, MS 39216, USA; 5 Department of Psychology, University of Kansas, 1450 Jayhawk Boulevard, Lawrence, KS 66045, USA

**Keywords:** Headache, Children, Migraine, Behavioral treatments, E-health, CD-ROM, Child, Migraine headaches, Cognitive-behavioral treatment

## Abstract

**Background:**

The purpose of this study was to evaluate the efficacy of a self-guided CD-ROM program (“Headstrong”) containing cognitive-behavioral self-management strategies versus an educational CD-ROM program for treating headaches, headache-related disability, and quality of life.

**Methods:**

Participants were 35 children ages 7–12 years with migraine recruited from one university medical center and two children’s hospital headache clinics. Participants were randomly assigned to complete the Headstrong or educational control CD-ROM program over a 4-week period. Data on headache frequency, duration, and severity, migraine-related disability, and quality of life (QOL) were obtained at baseline, post-intervention, and 3-months post-intervention.

**Results:**

At post-intervention, Headstrong resulted in lower severity (on a 10-point scale) than the control group by child report (5.06 ± 1.50 SD vs. 6.25 ± 1.92 SD, p = 0.03, ES = 0.7). At 3-months post-intervention, parents reported less migraine-related disability (on the PedMIDAS) in the Headstrong group compared to the control group (1.36 ± 2.06 SD vs. 5.18 ± 6.40 SD; p = 0.04, ES = 0.8). There were no other group differences at post treatment or at 3-months post-intervention.

**Conclusions:**

When compared to an educational control, Headstrong resulted in lower pain severity at post-treatment and less migraine-related disability at 3-months post-intervention, by child and parent report respectively. Headache frequency and quality of life did not change more for Headstrong versus control. Additional research is needed on the Headstrong Program to increase its efficacy and to test it with a larger sample recruited from multiple centers simultaneously.

## Background

Headaches are one of the most common pain conditions affecting children and adolescents. Worldwide, the prevalence of headache in children and adolescents has been estimated to be as high as 58.4%, with migraine occurring in up to 10% of young children [1]. Headaches can result in substantial morbidity, including a high frequency of school absences [2] and reduced overall quality of life [3]. Moreover, without early intervention, recurrences of headaches in childhood can contribute to more frequent and disabling headaches during adolescence and beyond [4].

Contemporary perspectives on the etiology and maintenance of primary headaches in children continue to be founded on a model that integrates genetic and biological predisposition with psychosocial context [5]. As such, integration of biobehavioral management with pharmacological interventions in a multidisciplinary treatment approach is regarded as the optimal standard of care for pediatric migraine [6,7]. Traditional biobehavioral interventions include relaxation, biofeedback, contingency management, and cognitive pain coping strategies. Such interventions are considered “empirically-supported treatments” [8,9], with meta-analyses supporting reliable reductions in pain associated with psychological therapies [10,11].

To broaden access to effective cognitive-behavioral treatment in children, CD-ROM and internet-based pain management programs have been developed and tested with children and adolescents with migraine [12-14] and other recurrent or persistent pain conditions [15-17]. In general, these studies have found positive outcomes forreducing pain relative to control conditions and the results are similar to those found in traditional biobehavioral interventions delivered in face-to-face contacts. Because of promising results in a pilot study of the Headstrong program tested against a wait-list control group in young children (ages 7–12) [12], the current randomized clinical trial was developed to evaluate this Headstrong CD-ROM intervention (identical to the pilot study version as outlined in Table 1) versus a more stringent control condition – one involving active educational content. The education condition controls for contact time and other non-specific therapeutic factors while also including active components of standardized education commonly provided in biobehavioral clinical care for pediatric headache [18,19]. We hypothesized that children with migraine headaches who received the Headstrong program would demonstrate greater improvement on headache outcomes (frequency, intensity, and duration), lower levels of headache-related disability, and better quality of life than children in the education control group.

**Table 1:** Participants’ characteristics and clinical baseline headache parameters

	**Educational control**		**Headstrong**		
	**(n = 17)**		**(n = 18)**		**p**
Demographics					
Age (yrs, *M*, *SD*)	10.2	1.5	10.2	2.0	0.91
Gender (female, *n*,%)	15	88%	10	56%	0.06
Ethnicity (Caucasian, *n*,%)	16	94%	17	94%	1.00
Grade in school (*M*, *SD*)	4.6	1.6	4.4	2.1	0.91
CBCL total score (*M*, *SD*)	52.0	7.8	49.9	11.0	0.60
Mother's age (yrs, *M*, *SD*)	35.7	6.2	37.9	8.1	0.50
Father's age (yrs, *M*, *SD*)	37.9	5.8	41.8	8.1	0.17
Mother's ed (some college, *n*,%)	11	65%	13	76%	0.71
Father's ed (some college, *n*,%)	11 69%	12	67%	1.00	
Family income (>$50,000, *n*,%)	10	59%	15	83%	0.15
Headache parameters					
Migraine diagnosis (*n*,%)	17	100%	16	89%	0.49
Ibuprofen prescribed (*n*,%)	8	47%	12	67%	0.32
Nortriptyline prescribed (n,%)	2	12%	4	22%	0.658

*Note*: p-value for difference between groups, based on Mann–Whitney U or Fisher's Exact test. M = mean, SD = standard deviation.

## Method

### Participants

Institutional Review Board approval was granted for this study at each recruitment site and informed consent was obtained from the parent(s) or caregiver(s) of each participant. A multi-center, randomized methodology was used with participants being recruited from pediatric headache clinics at one university medical center and two children’s hospitals in the mid-west between December 2004 and March of 2010. Participants were not run concurrently at all three sites, thus the reason for a six year study period. Participants were stratified by age (7–9 and 10–12) and randomly assigned following baseline to one of the two groups (education control or Headstrong). Participants met the following inclusion criteria: (a) 7–12 years of age; (b) having migraine occurring on the average at least once per week by parental or child report and separated by symptom-free periods; and (c) having a board-certified neurologist's diagnosis of migraine with or without aura, using International Classification of Headache Disorders [20]. Children were excluded from the study if (a) their medical history and/or neurological exam suggested that theirs were secondary headaches; (b) parents reported the child had been diagnosed with a mental health condition or was receiving concurrent psychotherapy; (c) scores on the internalizing or externalizing scales of the parent-reported Child Behavior Checklist [21] were in the clinical range at baseline; or (d) the baseline headache diaries indicated an average headache frequency of less than one per week (over a 14-day period). Participant descriptive statistics are shown in Table 2.

**Table 2:** Measures taken each week from beginning of study, by phase

**Phase**	**Baseline**		**Intervention**	**Post-intervention**		**3-months post-intervention**	
Study week:	1	2	3 to 6	7	8	19	20
Child behavior checklist and demographics	•		No measures taken				
Daily headache diary	•	•		•	•	•	•
PedsQL 4.0		•			•		•
PedMIDAS		•			•		•

### Research design

#### Control group

Children in the control group continued to follow the recommendations and prescriptions of their treating neurologist. Typically, treatment included acute medications (e.g., NSAIDs, triptans, and muscle relaxants) and/or preventative medications for children with a frequency of headache greater than one per week (e.g., anticonvulsants, antidepressants, beta-blockers).

Control participants received a developmentally appropriate educational CD-ROM program (see Table 1) containing information about primary headaches (i.e., types of primary headache, how headaches are assessed, typical symptoms, typical triggers, prevalence, etiology, and the multiple components of pain). The information contained in the education CD-ROM was more in-depth but similar to that contained in the first part of the Headstrong program (i.e., Module #1: Education). The education CD-ROM also covered health habits (e.g., sleep, diet, physical activity), but no “active” psychological headache therapies (e.g., relaxation, cognitive restructuring) were contained in this program. Children were asked to complete the program in 4 weeks, with approximately 1 lesson per day. The control CD-ROM controlled for the amount of headache education the two groups received and the time taken to completing the program. Parents also were given a manual containing directions on how to use the educational CD-ROM program, their role in the program (e.g., how to assist with homework assignments, how to complete headache diaries, etc.), lesson overviews, and technical assistance information in case their child had problems running the program. Children were asked to record passwords obtained at the end of each lesson; password sheets and homework assignment sheets were then returned by mail so that adherence to the program could be monitored.

#### Headstrong group

Participants in the Headstrong Group received the Headstrong CD-ROM program (see Table 1) while continuing to follow their treating neurologist’s recommendations and prescriptions. Children were asked to complete the program in 4 weeks, with approximately 1 lesson per day and were required to take simple quizzes to assess their processing of the information presented. As with the Control group, various passwords and homework assignments were embedded within the program to ensure that children were adherent in viewing and applying the material.

The layout of the cognitive-behavioral component of the CD-ROM intervention was similar to the education component of the Control group (including graphics, audio narration, music, clickable progress controls, passwords, homework assignments). However, the treatment component also contained lessons on how to use various empirically supported cognitive-behavioral treatments to self-manage recurrent headaches. Specifically, week two focused on relaxation methods (including a rationale with narrated and illustrated instructions on guided imagery, deep breathing, and progressive muscle relaxation), week three focused on problem-solving and stress management, and week four targeted pain behavior and parental response to pain as well as a review of the previous weeks' lessons. A workbook accompanied the Headstrong CD-ROM and contained all the supplementary material required for the self-management intervention. Parents were also given a manual containing directions on how to use the Headstrong program, their role in the intervention (e.g., how to assist with homework assignments, how to complete headache diaries, etc.), lesson overviews, and technical assistance information in case their child had problems running the program.

### Procedures

Once children and families provided informed consent, dependent measures were collected weekly via pre-paid mailers over a two-week baseline phase. Standard medical care was continued during baseline as throughout the study. If the child or parent had questions about headache activity or treatment during baseline, they were asked to speak with the treating neurologist and/or Headache Center Team. Contacts by research staff with children and parents were limited to weekly telephone calls to encourage consistent record keeping.

Following baseline, children were randomly assigned to the control or treatment group. Children in the control group were sent the educational CD-ROM, while those in the treatment group received the Headstrong CD-ROM. There were two versions of the educational and Headstrong CD-ROM program; one for participants 7–9 years and one for 10–12 year olds. Over the course of four weeks, Control and Treatment children worked through their respective lessons. Children were able to navigate through the different lessons at their own pace and were required to take simple quizzes to assess their processing of the information presented. Various passwords and homework assignments were embedded within the program to ensure that children were going through the material. Children were asked to record passwords obtained at the end of each lesson; password sheets and homework assignment sheets were then returned by mail so that adherence to the program could be monitored. Weekly phone calls continued during this intervention phase to answer questions about the CD-ROMs and to remind participants to complete and return password sheets. No measures were collected during the four-week intervention phase. Dependent measures were collected for two weeks immediately following the intervention via pre-paid mailers. Weekly phone calls continued during this two-week post-intervention phase to encourage consistent record keeping. Measures were then sent to families to be completed during the last two weeks of the 3-month post-intervention phase (see Table 2). Participants and their parents completed all measures independently.

#### Child behavior checklist and demographics

Parents completed the Child Behavior Checklist [21] before other baseline measures were obtained to determine if the child should be excluded from participation because of clinically significant elevations (above the 70^th^ percentile) on either the externalizing or internalizing scale. At the beginning of baseline, demographic data were obtained that included the child’s age, gender, ethnicity, grade in school, time since first experience of headache symptoms, involvement in psychological services, and current medications. We also collected information on the parents’ age, marital status, education, occupation, and income.

#### Headache diaries

Headache frequency, intensity/severity, and duration were reported on daily paper and pencil-based diaries. Just before going to bed, children recorded that day's headache occurrences (if any), severity and duration. Children reported whether or not they had a headache that day by circling yes or no. Two items were available each day in the event of more than one headache [22]. Children reported daily headache duration by recording the time a headache started and the time it stopped. Again, two items were available each day in the event of more than one headache, with the mean of the separate durations being used in analyses (minimum = 0; maximum = 14 hr). Daily headache severity was rated using a visual analog scale (VAS) ranging from 0 centimeters (no pain) to 10 centimeters (severe pain) [12]. The scale was illustrated with “pain face” anchors to help younger children rate their pain severity. Two scales were available to children each day, in the event of more than one headache, with the mean of the separate severities being used in analyses. Parents reported the child's daily headache occurrences, durations, and severities using the same measures as those used by the children.

#### Headache-related disability

The Pediatric Migraine Disability Assessment (PedMIDAS) is the only specific measure of headache-related disability in the pediatric population. The measure quantifies headache-related disability during the past three months across school, home, sports and social activities [23,24]. Responses are summed and then graded as reflecting little to none (0 to 10, Grade I), mild (11 to 30, Grade II), moderate (31 to 50, Grade III), and severe disability (greater than 50, Grade IV) [25]. PedMIDAS correlates significantly with frequency, duration, and severity of headache and is sensitive to treatment response. PedMIDAS total score ranges from 0 (no headache-related disability) to anything over 50 (severe headache-related disability [maximum score of 240].

#### Quality of life (QOL)

We used the fourth edition of the Pediatric Quality of Life Inventory [26,27]. PedsQL 4.0's 23 items require children to report on various aspects of physical functioning (8 items), emotional functioning (5 items), social functioning (5 items), and school functioning (5 items). Respondents indicate the extent to which they are having problems in each of these areas using a 0 (“never a problem”) to 4 (“almost always a problem”) response scale. Items are then reverse-scored and linearly transformed to a 0–100 scale such that higher scores indicate better quality of life. A parent-report version of PedsQL 4.0, identical to the child self-report version, was also administered to obtain the parents’ perspectives on their children’s quality of life. This scale has excellent psychometric properties [28-30] and it can reliably document the effects of pediatric migraine on QOL [3,31]. The Total Scale Score has minimum = 0 (poorest quality of life) and maximum = 100 (excellent quality of life).

### Statistical analyses

**Figure 1:**
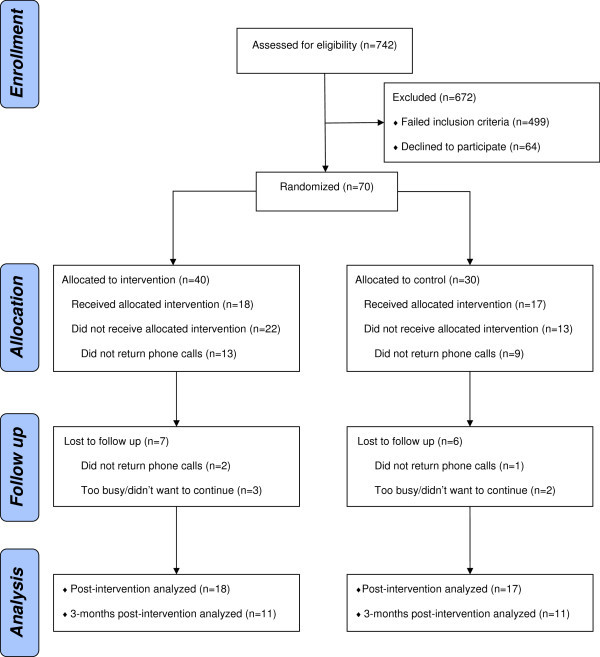
Consort diagram showing recruitment and retention of participants.

Sample size calculation was based on improvements in headache severity going from baseline to post-intervention. The model utilized the Groups × Phases interaction in a 2 × 2 RM ANOVA with the anticipated effect being a mean decrease of at least 1 point (10-point analog scale) for the Headstrong group versus no change for the control group. Baseline values were estimated to be mean (SD) 5.0 (1.6) by pooling the values from three relevant studies [12-14]. Seeking 80% power for the interaction and assuming a two-tail alpha = 0.05 and a 0.60 correlation between baseline and follow up, nQuery Advisor 7.0 (Statistical Solutions, Saugus, Mass.) indicated n = 34 per group, total N = 68. Assuming 15% attrition, the final target value was set to n = 40 per group, total N = 80. The final group sizes fell far short of those required by the sample-size determination (see Figure 1). Statistical analyses were therefore necessarily restricted to univariate tests of differences between the two groups. There was insufficient power to correct for multiple comparisons or to covary for potential confounding variables. Demographics are thus reported only for descriptive purposes. With p < 0.05 we ran one-tailed tests of treatment effects on the headache measures and we augmented interpretation of the observed *p*-values with the corresponding effect sizes (Cohen's ES = |M_1_-M_2_| ÷ SD _pooled_; [32]). Regardless of the associated p-value, ESs between 0.50 and 0.80 were considered to be in the medium to large range and therefore worthy of provisional discussion.

## Results

### Participants

The CONSORT diagram (see Figure 1) shows the number of clinic visits and number of potential participants who were screened, the number of participants randomized, and the number who completed the protocol through at least the post-interventions phase (Headstrong n = 18; Control n = 17). We were required, by the IRB at one site, to purge 24 participants from the database because of consenting errors, most of which involved the witness and parent signing the form on different dates (i.e., the parent had asked to take the consent form home and was called by the research assistant and consented over the phone, but the form was mailed back and therefore not witnessed by signature until some few days later). The final sample was primarily Caucasian (94%) with family income generally over $50,000 (71%), both parents having at least some college education (mothers 71%, fathers 68%), and the children diagnosed most frequently with migraine headache (94%) and treated with prescribed ibuprofen (57%) and/or nortriptyline (17%). Table 3 shows the breakdown of these values by group.

**Table 3:** CD-ROM lessons for education control and headstrong group

	**Educational control lessons**	**Headstrong lessons**
Week 1	**Headache education**	**Headache education & cognitive-behavioral model of pain**
	1). Introduction	1). Introduction
	2). Types of headache	2). Types of headache
	3). Prevalence of headache	3). Prevalence of headache
	4). Features of headache	4). Features of headache
	5). How headache is diagnosed	5). How headache is diagnosed
		6). The pain puzzle
		7). Headache triggers
Week 2	**Cognitive-behavioral model of pain**	**Relaxation**
	1). Introduction to the pain puzzle	1). Rationale for relaxation
	2). puzzle piece 1: Nociception	2). How to use guided imagery
	3). Puzzle piece 2: Thoughts	3). How to use deep breathing
		4). How to use progressive muscle relaxation
Week 3	**Cognitive-behavioral model of pain**	**Cognitive restructuring**
	1). Puzzle piece 3: Feelings	1). Rationale for coping
	2). Puzzle piece 4: Behavior	2). Thought-changing
		3). Problem-solving
Week 4	**Headache triggers**	**Pain behaviors**
	1). Introduction to headache triggers	1). Positive and negative pain behaviors
	2). Key headache triggers: diet and sleep	2). Importance of keeping active
		3). Review of all lessons

### Child reported outcomes

Complete results for the child reported outcome measures are shown in Table 4, including the results of comparisons between the education control and Headstrong groups. The only statistically significant between-group difference at post-intervention was in pain severity, with the Headstrong group reporting significantly lower pain severity than education controls (M = 5.06 vs. 6.25; p = 0.03; ES = 0.7). While moderate effects (ES = 0.70) were also found for headache duration and disability at 3-months post-intervention favoring the Headstrong group (see Table 4), these differences were not statistically significant.

**Table 4:** Children's results for headache outcomes, PedMIDAS, and PedsQL 4.0 at baseline, post-intervention and 3-months post-intervention

		**Baseline**		**Post-intervention**		**3-Months post-intervention**	
		**TX**	**CTRL**	**TX**	**CTRL**	**TX**	**CTRL**
	n	18.. .	17. . .	18. . .	17. . .	11. . .	11. . .
**Headache frequency (% of days)**	Mean	41.09	40.67	31.28	32.14	21.43	18.18
	Median	35.71	35.71	25.00	28.57	7.14	14.29
	SD	22.67	28.79	28.24	22.23	23.47	17.60
	p	0.48		0.46		0.36	
	ES	0.0		0.0		0.2	
**Headache duration (hr/episode)**	Mean	5.47	4.15	4.47	5.56	1.53	4.25
	Median	4.04	2.86	3.71	4.25	1.50	2.65
	SD	4.20	3.88	4.26	4.01	0.91	5.19
	p	0.19		0.24		0.07	
	ES	0.3		0.3		0.7	
**Headache severity (10-point VAS)**	Mean	5.06	6.00	5.06	6.25	4.46	3.68
	Median	4.61	5.90	5.42	6.06	4.33	3.75
	SD	1.84	1.52	1.50	1.92	1.88	2.04
	p	0.07		0.03*		0.20	
	ES	0.6		0.7		0.4	
**PedMIDAS total (0 to >50)**	Mean	13.26	15.53	7.82	12.29	0.91	3.50
	Median	14.00	12.00	3.00	7.00	0.00	2.00
	*SD*	9.69	10.08	10.59	12.94	1.45	4.86
	p	0.25		0.14		0.05	
	ES	0.2		0.4		0.7	
**PedsQL total (0 to 100)**	Mean	82.10	79.35	83.70	80.69	84.88	85.67
	Median	82.61	83.70	85.87	84.78	93.48	89.13
	*SD*	12.18	11.55	12.07	14.36	18.22	14.32
	p	0.25		0.26		0.46	
	ES	0.2		0.2		0.0	

*Note:* TX = treatment group, Headstrong; CTRL = educational control group; n = number of participants; ES = Effect size (Cohen's *d*) for difference between independent means. A value of 0.5 or greater indicates a medium effect size and 0.8 or greater a large effect size; p = 1-tail significance of the difference between the two means directly above it. *A value of < 0.05 indicates a significant difference in means.

### Parent reported outcomes

Complete results for the parent reported outcome measures are shown in Table 5, including the results of comparisons between the Control and Headstrong groups. The only significant between-group difference was on PedMIDAS at 3-months post-intervention, with the Headstrong group having significantly less disability compared to educational controls (means = 1.36 vs. 5.18; p = 0.04; ES = 0.8). While a large effect (0.8) was also found for headache duration in favor of the Headstrong group at 3-months post-intervention (see Table 5), this difference was not statistically significant. Note, however, the much-reduced sample size (n = 22) at 3-months post-intervention.

**Table 5:** Parent results for headache outcomes, PedMIDAS, and PedsQL 4.0 during baseline, post-intervention and 3-months post-intervention

		**Baseline**		**Post-intervention**		**3-Months post-intervention**	
		**TX**	**CTRL**	**TX**	**CTRL**	**TX**	**CTRL**
	n	18…	17…	18…	17…	11…	11…
**Headache frequency (% of days)**	Mean	39.29	38.66	30.93	30.25	19.48	16.88
	Median	35.71	35.71	25.00	28.57	7.14	14.29
	*SD*	22.35	28.80	28.74	21.51	22.61	17.58
	p	0.47		0.47		0.38	
	ES	0.0		0.0		0.1	
**Headache duration (hr/episode)**	Mean	5.69	3.72	5.63	5.25	1.76	3.94
	Median	3.65	2.46	4.29	4.19	1.50	4.25
	*SD*	4.58	3.49	4.17	3.58	1.23	3.95
	p	0.10		0.40		0.08	
	ES	0.5		0.1		0.8	
**Headache severity (10-point VAS)**	Mean	6.37	6.52	6.15	6.84	4.57	5.63
	Median	6.46	6.77	6.25	7.19	4.75	5.00
	*SD*	2.01	1.92	1.56	1.37	1.98	2.30
	p	0.42		0.10		0.17	
	ES	0.0		0.5		0.5	
**PedMIDAS total**	Mean	14.94	14.21	9.06	10.50	1.36	5.18
**(0 to >50)**	Median	15.00	12.50	4.00	6.00	0.00	2.00
	*SD*	10.63	8.41	11.82	11.24	2.06	6.40
	p	0.41		0.36		0.04	
	ES	0.1		0.1		0.8	
**PedsQL total**	Mean	78.07	80.95	81.01	81.84	88.74	88.73
**(0 to 100)**	Median	77.17	79.35	82.61	84.78	93.48	91.30
	*SD*	14.17	9.81	12.36	15.95	11.28	7.75
	p	0.25		0.43		0.50	
	ES	0.2		0.1		0.0	

*Note:* TX = treatment group, Headstrong; CTRL = educational control group; n = number of participants; ES = Effect size (Cohen's *d*) for difference between independent means. A value of 0.5 or greater indicates a medium effect size and 0.8 or greater a large effect size; p = 1-tail significance of the difference between the two means directly above it. A value of < 0.05 indicates a significant difference in means.

## Discussion

Given the small number of participants who were eligible and completed this randomized controlled-trial, one could view the results as a second pilot study of the Headstrong program using a control condition (educational control) more stringent than the wait-list control used in the first plot study [12]. In the first study, significant differences were observed between the groups favoring Headstrong in headache frequency, severity, and duration, but there were no differences in headache disability (PedMIDAS). In the current study, there were statistically significant differences in favor of the Headstrong only for severity at post-intervention by child ratings and for PedMIDAS at 3-months post-intervention by parent ratings. Though not statistically significant, medium effect sizes favored the Headstrong group for headache duration and PedMIDAS at 3-months post-intervention by child ratings (both having ES = 0.70). Effect size also favored the Headstrong group on headache duration at 3-months post-intervention by parent ratings (0.80, or in the large range) even though the p-value = .08 was not significant. These differences in favor of the Headstrong group might have been significant were the groups as large as those specified in the study's sample-size calculations but this cannot be proven in the current study. Several differences between this study and the original one, such as measurement differences, differences in study timeline, and analytic differences, make for difficult direct comparisons of the results.

This study has several major limitations. First, the study was under-powered according to pre-determined sample size requirements. Generalizability of these findings is also limited for the broader pediatric migraine population because of our exclusion of children with headaches less than once per week and exclusion of children with mental health conditions, which may lead to sample biases. These limitations are somewhat tempered by our recruitment of children and families from three different medical centers. Any follow-up study must address the concern of recruitment size by conducting a multi-site effort. Additional techniques such as more regular and positive contact with participants, creative incentives, reducing participant burden, and having improved tracking systems may also help minimize attrition in the future [33]. In addition to the limitations of sample size, the educational control CD-ROM program had some potentially active treatment elements, such as information on diet, sleep, and avoidance of headache triggers. Our plans for the future are to modify the education control program to avoid discussion of factors that are known to mitigate headaches, such as emotional, behavioral, and stress triggers. Finally, for the Headstrong group the baseline PedMIDAS mean score was low (*M* = 13.3; considered to be Grade II, mild disability [25] while their baseline PedsQL 4.0 mean score was high (82.10), which is close to the mean of 83 found for a healthy sample [30]. Thus, a favorable change on either of these secondary measures was potentially limited by its proximity to the scale limit.

Despite the study's limitations, the effect sizes reported here and results of our previous pilot study combine to suggest that the Headstrong program may positively influence children's headaches by providing education about headaches and cognitive-behavioral skills in managing headache symptoms. The Headstrong program may also decrease headache-related disability over the long-term by providing children with additional tools for coping with migraines and chronic tension-type headaches. In view of the results of this study and the initial pilot study [12], we would recommend the use of the Headstrong program in clinical settings as an initial adjunctive treatment for pediatric headaches in combination with medications. Some patients may require additional face-to-face cognitive-behavioral treatment if Headstrong does not produce acceptable levels of improvement in headache frequency and/or severity.

## Conclusions

When compared to an educational control, Headstrong resulted in lower pain severity at post-treatment and less migraine-related disability at 3-months post-intervention, by child and parent report respectively. Headache frequency and quality of life did not change more for Headstrong versus control. Additional research is needed on the Headstrong Program to increase its efficacy and to test it with a larger sample recruited from multiple centers simultaneously.

## Competing interests

The authors declare that they have no competing interests.

## Authors’ contributions

MAR was the principal investigator for this study and was lead author on this paper; MC also took a lead role in writing this paper and recruited subjects for the study at his site; JLB diagnosed and referred subjects to the study and contributed to editing of the paper; SWP supervised recruitment of subjects and data collection at his site and made editorial revisions to the paper; ADH diagnosed and referred subjects to the study and made editorial revisions to the paper; JRA was a research coordinator and was responsible for recruitment, retention, data collection, data entry, and regulatory management at the Cincinnati Site and made editorial revisions to the paper; CWK and CCL served as research assistants and were responsible for recruitment, data collection, and data entry at the Kansas site; JMB did the statistical analyses and write-up for this study and made substantial editorial contributions to the paper. All authors read and approved the final manuscript.
